# Incidental Findings on Cone Beam Computed Tomography Studies outside of the Maxillofacial Skeleton

**DOI:** 10.1155/2016/9196503

**Published:** 2016-07-04

**Authors:** Sevin Barghan, Mehrnaz Tahmasbi Arashlow, Madhu K. Nair

**Affiliations:** ^1^Department of Oral and Maxillofacial Diagnostic Sciences, University of Florida Colleges of Dentistry and Medicine, 1395 Center Drive, Room D8-6, Gainesville, FL 32610, USA; ^2^Department of Oral and Maxillofacial Diagnostic Sciences, Oral and Maxillofacial Radiology, University of Florida Colleges of Dentistry and Medicine, 1395 Center Drive, Room D8-6, Gainesville, FL 32610, USA; ^3^Department of Oral and Maxillofacial Diagnostic Sciences, University of Florida Colleges of Dentistry and Medicine, 1395 Center Drive, Room D8-6D, Gainesville, FL 32610, USA

## Abstract

*Objective*. To define the presence and prevalence of incidental findings in and around the base of skull from large field-of-view CBCT of the maxillofacial region and to determine their clinical importance.* Methods*. Four hundred consecutive large fields of view CBCT scans viewed from January 1, 2007, to January 1, 2014, were retrospectively evaluated for incidental findings of the cervical vertebrae and surrounding structures. Findings were categorized into cervical vertebrae, intracranial, soft tissue, airway, carotid artery, lymph node, and skull base findings.* Results*. A total of 653 incidental findings were identified in 309 of the 400 CBCT scans. The most prevalent incidental findings were soft tissue calcifications (29.71%), followed by intracranial calcifications (27.11%), cervical vertebrae (20.06%), airway (11.49%), external carotid artery calcification (10.41%), lymph node calcification (0.77%), subcutaneous tissue calcification and calcified tendonitis of the longus colli muscle (0.3%), and skull base finding (0.15%). A significant portion of the incidental findings (31.24%) required referral, 17.76% required monitoring, and 51% did not require either.* Conclusion*. A comprehensive review of the CBCT images beyond the region of interest, especially incidental findings in the base of skull, cervical vertebrae, pharyngeal airway, and soft tissue, is necessary to avoid overlooking clinically significant lesions.

## 1. Introduction

Cone beam computed tomography (CBCT) is a relatively common imaging modality widely used in maxillofacial imaging. In addition to dental abnormalities, CBCT also enables evaluation of anatomic structures such as maxillofacial bony structures, temporomandibular joints (TMJs), paranasal sinuses, upper cervical vertebrae, and the base of skull and pharyngeal airway. Other studies have reported incidental findings on CBCT [1–4]. An incidental finding is one that is discovered during the normal course of interpretation of a study and is unrelated to the condition that prompted the test. Studies have reported on incidental findings on CBCT related to the maxillary sinuses, dentition, TMJs, and/or localized manifestations of systemic disease [1, 3–12]. The primary objective of our study was to report on findings in other areas outside of the maxillofacial skeleton that are visualized on medium and large field-of-view (FoV) CBCT studies, given the relatively high spatial resolution achieved with the modality.

Large (FoV) CBCT studies can also reveal the normal and pathologic findings associated with osseous structures of the cervical spine and the base of skull, as well as some soft tissue entities. Recognition of major anatomic landmarks and radiographic features of incidental findings as visualized on CBCT studies is critical to the interpretation process. In addition to being a medicolegal issue, appropriate management of incidental findings can prevent undesirable outcomes in these patients. Findings, based on their location, nature, and effects on adjacent structures, may or may not require referral, intervention, more imaging, or long-term monitoring [13–16].

This study evaluated the prevalence and nature of incidental findings in and around the base of skull including but not limited to the cervical spine, lateral neck region, pharyngeal airway, and intracranial structures in CBCT studies which are usually reviewed by dentists and dental specialists.

## 2. Material and Methods

The study protocol was reviewed and approved by University of Florida Institutional Review Board (IRB). The images of 400 consecutive large FoV CBCT studies were reviewed in the Oral and Maxillofacial Radiology Clinic at the University of Florida, College of Dentistry from January 1, 2007, to January 1, 2014, for incidental findings related to the cervical spine, paravertebral regions, lateral neck region, pharyngeal airway, and intracranial structures. The chosen sample was deemed appropriate in size by comparison with similar studies in the literature. The images were viewed with InVivo Dental software (Anatomage, Inc., San Jose, CA, USA). The CBCT studies included in this study were the iCAT*™* (Imaging Sciences International, Hatfield, PA, USA), CS 9300 (Carestream Health, Atlanta, GA), NewTom QR-DVT-9000 (QR-NIM s.r.l., Verona, Italy), and the CS9500 (Carestream Health, Atlanta, GA). Exposure parameters include 70–125 kVp and 12–40 mAs, with differences in the fields of view and acquisition voxel sizes. Patients' demographic data, indications for imaging, and the types of CBCT units used were recorded. Findings related to the dentition, periodontium, paranasal sinuses, and temporomandibular joint diseases were not recorded. Findings directly related to the primary indications for CBCT scans were excluded. Images with poor image quality were also excluded. Diagnoses were based entirely on radiographic appearance using well-established radiographic interpretation processes. In radiology, incidental findings are defined as an occult entity discovered unexpectedly on an imaging examination performed for an unrelated reason [5]. The cervical vertebrae were evaluated for the presence of degenerative changes, malalignment, fusion, loss of intervertebral space, and any other pathoses. The airway was evaluated for the presence of narrowing, asymmetry, masses, and lymphoid hyperplasia. The neck region was investigated for the presence of any masses, asymmetry, lateralization or narrowing of the pharyngeal airway, or dystrophic calcification. In addition, the clivus, base of skull, and intracranial anatomy were evaluated for any abnormality. All studies had reports dictated earlier by board-certified oral and maxillofacial radiologists. However, this study involved evaluation of the datasets by two oral and maxillofacial radiology residents in their second and third years of training via independent sessions and under optimal viewing conditions. Findings were tabulated and compared with those reported by the radiologists. All cases with intracranial findings had been reviewed by board-certified neuroradiologists with head and neck fellowship training as well. Any additional findings or confounders observed by the residents were brought to the attention of the oral and maxillofacial radiologist and neuroradiologists as part of their training process. No additional findings were reported. Residents were included in the process to assess the extent to which nonradiologists would potentially miss incidental findings. After reviewing the types and prevalence of incidental findings in the regions of interest, findings were divided into three categories: those that required appropriate referral to a physician or specialist, those that required follow-up in the form of continued monitoring, and those that required no referral, follow-up, or monitoring but required documentation.

Following data collection, data analysis was performed with the help of Statistical Package for Social Sciences (SPSS) version 22. Qualitative analysis was done.

## 3. Results

**Figure 1:**
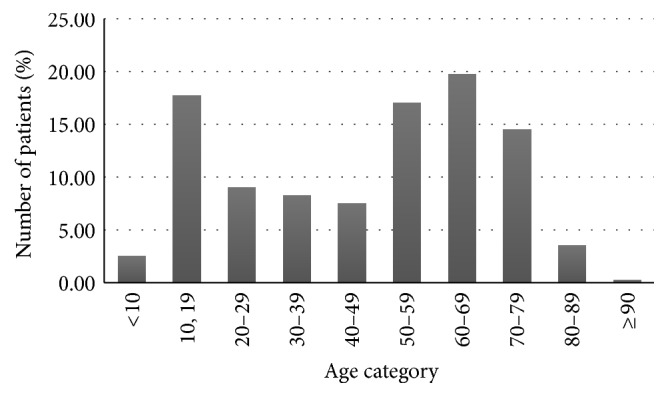
Age distribution of patients.

**Figure 2:**
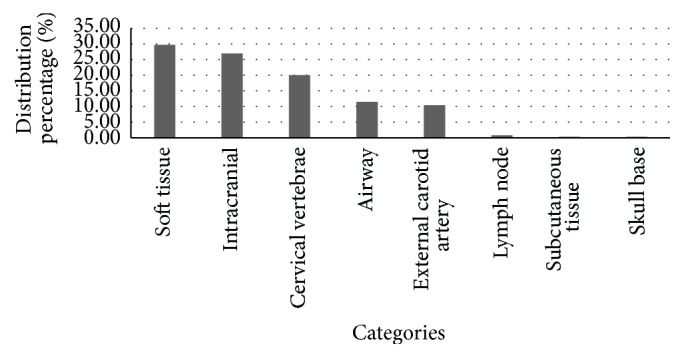
Distribution of findings.

Out of 400 patients, 146 (36.5%) were males and 254 (63.5%) were females. Mean age of patients referred for CBCT was 47.08 years. Most frequently referred patient age groups were the 60–69 age group (19.75%) (Figure 1). Patients were referred for orthodontic intervention (*n* = 96), implant treatment planning (*n* = 152), TMJ evaluation (*n* = 31), and evaluation of suspected pathoses (*n* = 121). The indications for CBCT examination are shown in Table 1. A total of 653 incidental findings were identified in 309 of the 400 CBCT studies (77%), representing an overall rate of 1.63 incidental findings per study. Ninety-one (22.75%) scans showed no incidental findings of relevance. The most prevalent incidental findings included soft tissue calcifications (29.71%), followed by intracranial calcifications (26.96%), cervical vertebral pathoses (20.06%), airway findings (11.49%), external carotid artery calcifications (10.41%), lymph node calcifications (0.77%), calcifications in subcutaneous tissue, calcific tendonitis of the longus colli muscle (0.3%), and skull base lesions (0.15%) (Figure 2).

Table 2 further subdivides these categories into more specific categories. A significant portion (31.39%) of the incidental findings required referral, 17.76% required monitoring, and 50.85% did not require either (Table 3).

## 4. Discussion

A total of 653 incidental findings were identified in 309 studies representing an overall rate of 2.1 incidental findings per scan. It is known that the frequency of incidental findings in CBCT imaging varies widely among studies in the literature, ranging from 1.1 to 2.9 incidental findings per CBCT scan. This is due to differences in age groups, demographics of patient studies, and categories of findings that were reported.

In this study, soft tissue calcifications represented the most frequent incidental finding (29.71%): thyroid cartilage/triticeous cartilage calcification (16.24%), tonsilloliths (8.42%), and stylohyoid/stylomandibular ligament calcification (5.05%). Calcifications in the various structures of the head and neck region are a relatively common finding. These calcifications occur as a result of either physiological (age-related) or pathologic mineralization. Soft tissue calcifications could be identified correctly based on anatomic location, morphology, and distribution [6]. The prevalence of the triticeous cartilage calcification in the literature was 12–65% [17, 18].

The wide range in observations results is likely due to the spatial resolution of the modality as compared to multidetector computed tomography studies, variable ossification patterns of the triticeous cartilage, and variability in the morphology, size, and position of this structure within the thyrohyoid ligament [19]. No treatment is required for calcified thyroid and triticeous cartilages [6].

**Figure 3:**
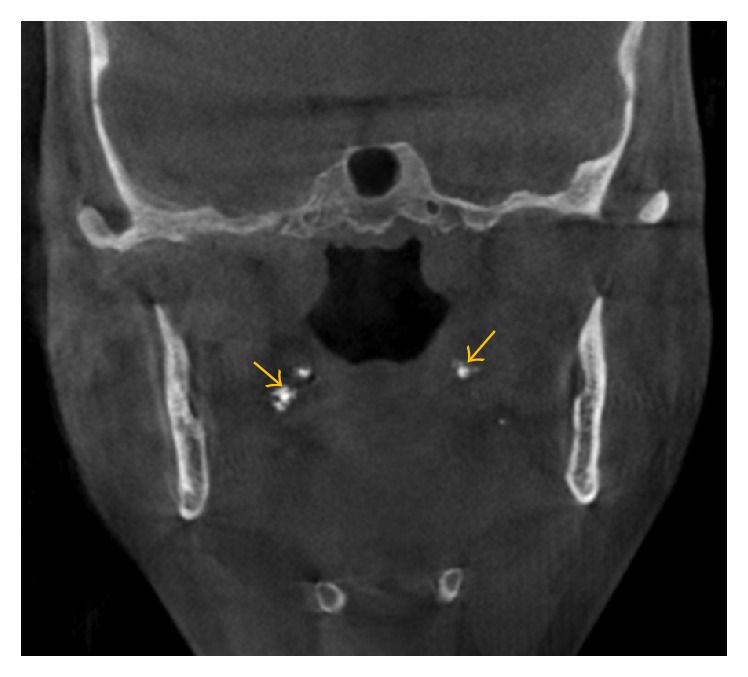
A coronal view shows tonsilloliths within the tonsils.

Tonsilloliths are common incidental findings, identified within 16–24% of patients (Figure 3) [20, 21]. Some studies suggest that tonsilloliths are clinically related to halitosis and prior history of repeated tonsillitis in childhood and/or tonsillar abscesses. Patients with tonsilloliths have a tenfold increase in the incidence of halitosis [20, 22–24]. No treatment is required for most tonsillar calcifications. However treatment may be considered in elderly patients with immunosuppression because of aspiration pneumonia [6].

Ossification of stylohyoid/stylomandibular ligament was 5.05% of all incidental findings. Reviewing different studies showed that approximately 4% of the general population has calcification of the stylohyoid ligaments, partial or complete, continuous or segmental. No follow-up or intervention is required unless the patient presents with symptoms associated with Eagle's syndrome [6].

**Figure 4:**
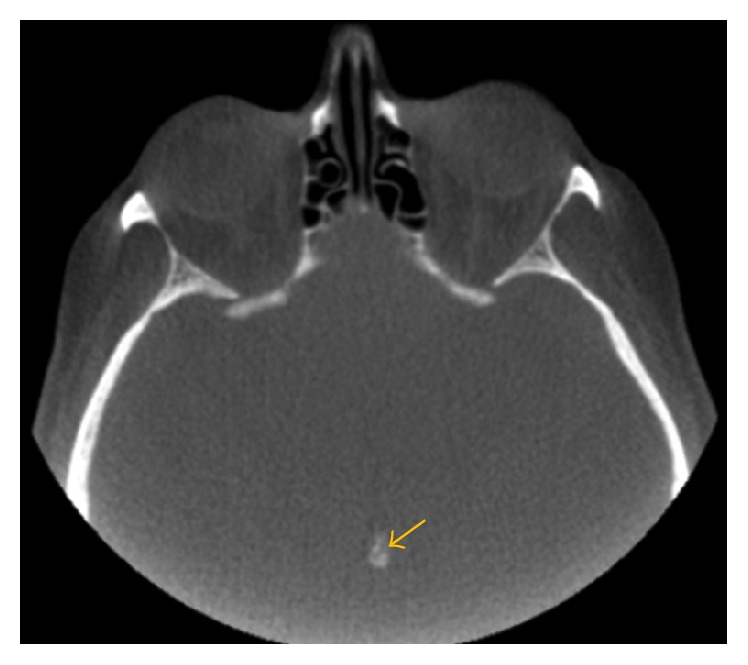
Axial image shows pineal gland calcification in the midline.

**Figure 5:**
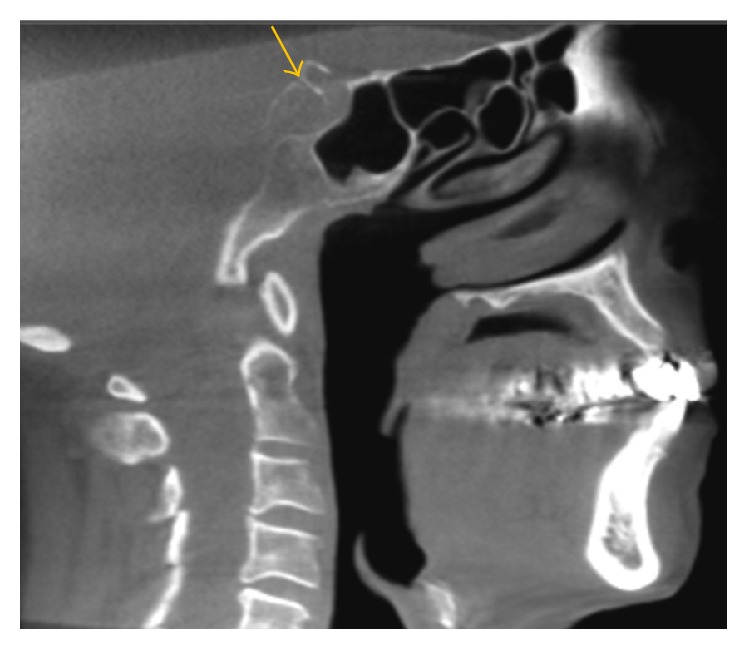
A sagittal view demonstrates intracranial vascular calcification.

The next most common incidental finding was intracranial calcifications (26.96%), mostly in the pineal gland (11.18%), followed by intracranial atherosclerosis (10.57%) (Figures 4 and 5).

Intracranial physiological calcifications can be a common incidental finding on studies in older patients being an age-related change [25–27]. Further evaluation is recommended if the patient has other risk factors for cerebrovascular accidents. Referral to the patient's physician is advised for additional imaging such as CTA (Computed Tomography Angiogram) for evaluation of intracranial atherosclerosis or other vascular anomalies.

The most common sites for physiologic calcifications are pineal gland, habenula, choroid plexus, basal ganglia, falx, tentorium, petroclinoid ligaments, and sagittal sinus. Calcification of the pineal gland is seen in two-thirds of the adult population and increases with age [28].

Pineal gland calcification was identified in 19.2% of CBCT subjects by Pette et al. [7] and in 13.1% by Admassie and Mekonnen [29]. Physiologic calcification is asymptomatic and is detected incidentally on advanced imaging. They are almost never clinically significant and often do not lead to any clinical concern [7, 29]. Pineal gland calcifications are rare in children younger than 6 years of age. The possibility of pineal gland tumor should be considered when the calcification is found in the children younger than 9 years or when it is greater than 1 cm in diameter [30].

Intracranial vascular atheroscleroses were reported by Pette et al. [7] in 23.6% of their subjects. Other studies identified internal carotid artery (ICA) calcifications in 4.8% and 5.7% of subjects [6, 8].

The presence of ICA calcifications does not always imply stenosis. The gold standard for the diagnosis of carotid artery stenosis (CAS) is Doppler ultrasound [31, 32]. Identifying intracranial carotid calcification is important and physician referral is recommended for evaluation of risk factors for stroke [32–36].

In our study, one case with some atherosclerosis in the right carotid tree area was noted with evidence of extensive calcifications within the Circle of Willis as also suspected dilatation of the posterior communicating artery [37]. The patient was asymptomatic and had reported for removal of a benign tumor of the mandible (keratocystic odontogenic tumor). Suspected findings on the CBCT study prompted further evaluation of the patient's history. On further questioning, patient reported that her mother had died from a ruptured aneurysm at around the age of 55. Multidetector computed tomography (MDCT) and CT Angiography (CTA) were performed on this patient (53-year-old Caucasian female), which confirmed the presence of a large aneurysm in the posterior communicating artery within the Circle of Willis, as suspected on the CBCT. A stat neurosurgery consult determined that the lesion was life-threatening owing to its size and relative thinning of the vessel walls, resulting in a craniotomy procedure being performed immediately. Even though CBCT is known to be suboptimal for detection of soft tissue pathoses, careful and sequential evaluation of CBCT slices using appropriate postprocessing filters and slice thicknesses is advised for all studies of the head and neck region to possibly tease out suspicious entities that merit further evaluation including advanced imaging studies.

The incidence of airway findings was 11.49%. The major types of findings in the airway were pharyngeal airway narrowing (6.9%), followed by adenoid hypertrophy (3.52%) and asymmetry of pharyngeal airway (1.07%). Other CBCT studies have demonstrated that airway findings represent 8.4% to 35.0% of total CBCT findings [6, 7, 9].

**Figure 6:**
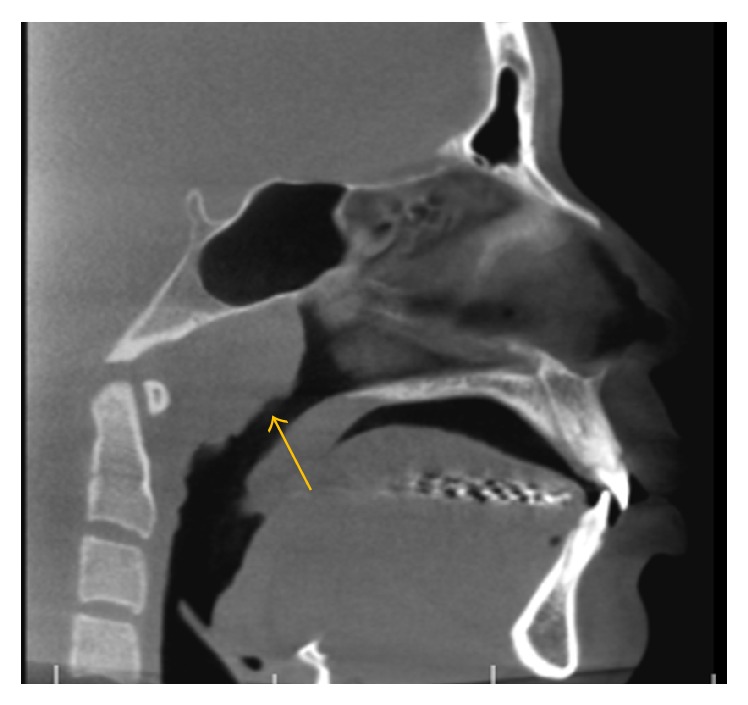
A sagittal view demonstrates marked adenoidal hyperplasia in a 16-year-old female.

Narrowing or asymmetry of the pharyngeal airway may be associated with obstructive sleep apnea or benign or malignant tumors originating in any of the adjacent head and neck spaces or the base of tongue. The exact cause of airway narrowing and asymmetry cannot be established based on CBCT alone owing to lack of adequate visualization of soft tissue entities. Physician referral is essential, following correlation with patient history and/or clinical findings [38]. Physiological adenoid hypertrophy is common in children between the ages of 6 and 10 years, following which atrophy sets in by the age of 16 years [39]. In our study mean age of adenoid hypertrophy was 13 years old (Figure 6). CBCT can be an important tool in the initial assessment of suspected airway abnormalities. An important distinction must be made between identifying potential airway constriction in CBCT imaging and the actual presence and/or severity of clinical obstruction [10]. No definitive quantitative assessment of compromise in airway volume must be made solely based on CBCT.

Our study reports 20.06% of incidental findings in the cervical vertebral region. This is in contrast to CBCT studies by Pliska et al. [9], Edwards et al. [10], and Drage et al. [11], all of which included cases with lower mean age in a patient population seeking treatment for malocclusion. In these studies, cervical vertebral findings were identified in merely 1.3% of cases.

**Figure 7:**
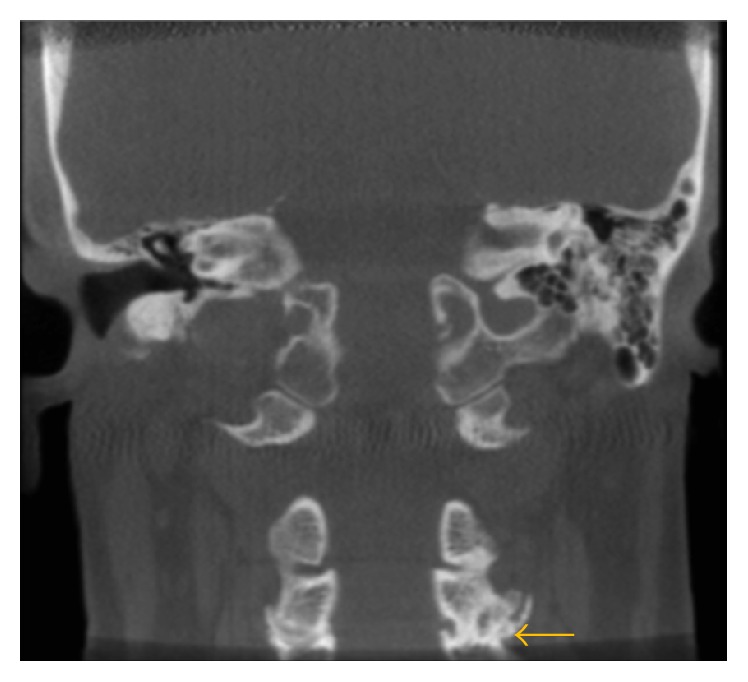
Degenerative changes (osteoarthritis) of the C3-C4 with osteophyte formation are seen on this coronal image.

CBCT studies by Pette et al. [7] and Allareddy et al. [8] identified cervical vertebral findings in 47.8% and 9.7% of subjects, respectively, with degenerative changes representing the majority of findings. In our study the main findings in cervical vertebra region were degenerative changes as well (16.38%) (Figure 7).

**Figure 8:**
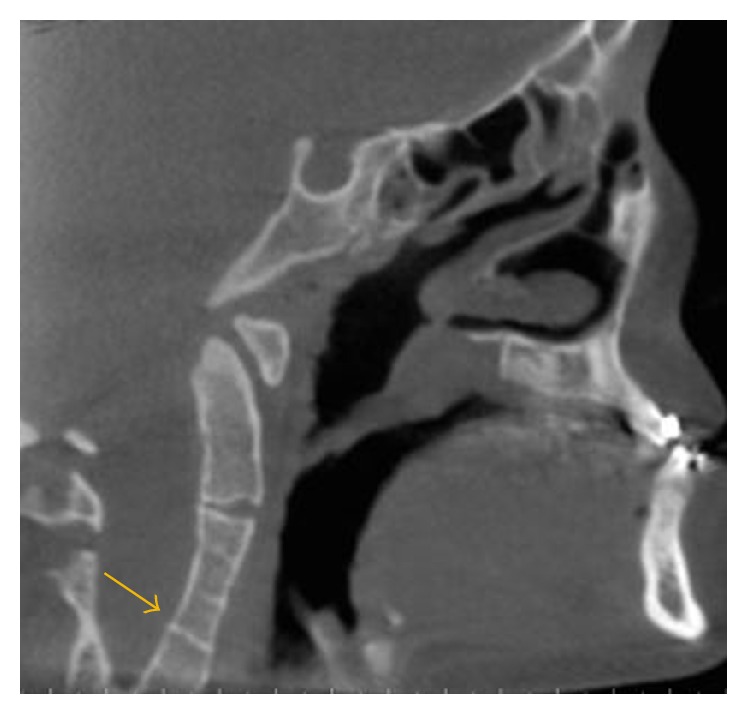
A sagittal view demonstrates fusion of the C3-C4.

In addition to degenerative changes, other findings were identified in our study including well-defined and delineated lytic areas (1.38%), vertebral misalignment (0.91%), and fusion (0.77%). The prevalence of vertebral fusion in other studies is 0.4 to 0.7% (Figure 8) [10, 40].

Monitoring of the degenerative changes of the cervical vertebrae is recommended to assess whether there are any indications for further referral and/or intervention [41]. Lytic lesions in the cervical vertebrae include pneumatocysts, other gas-containing lesions such as those seen in osteomyelitis, osteonecrosis, neoplasms such as hemangiomas, posttraumatic lesions, and degenerative lesions. Clinical evaluation by a physician is recommended in order to assess the need for additional imaging including MDCT and/or MRI (Magnetic Resonance Imaging) based on clinical findings and history. Some lesions such as intrabony hemangioma/s may require monitoring in future studies. If risk of enlargement of the lesion exists, radiographic follow-up is prudent [42–44]. Two patients had radiolucent lesion in the cervical vertebrae. Differential diagnoses must include invasive and erosive lesions, both benign and malignant. Pseudotumoral lesions, metastatic lesions, and hematologic malignancies must also be considered following correlation with clinical findings including neurologic deficits and assessment of history.

It is important to keep in mind that the cervical spine is the common site for cancer metastasis. Correlation of radiographic findings with medical history of primary cancer is of particular importance when determining the nature of bone density changes in the cervical vertebrae [45].

In addition to metastatic lesions in the cervical vertebrae, multiple myeloma presents with well-defined, punched-out lesions which usually involve multiple vertebral bodies. Unifocal and multifocal lesions must be considered. Central hemangiomas are also noted within the vertebrae. These can eventually lead to pathologic fractures as the patient ages and osteoporosis sets in or the hemangiomas grow. Referral is always recommended for better evaluation of the signals of interest and/or further advanced imaging.

**Figure 9:**
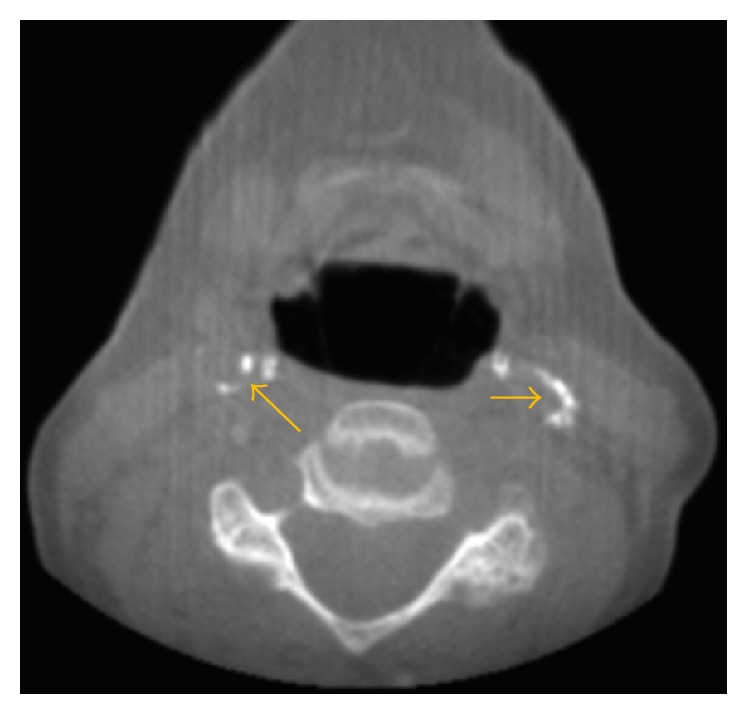
Carotid artery calcifications are seen bilaterally on this axial image.

Extracranial carotid artery calcification was noted in 10.41% of incidental findings (Figure 9). These calcifications are radiographic evidence of atherosclerosis and could be a risk indicator for potential stroke or metabolic disease. Our findings regarding prevalence of age-related carotid artery calcifications are similar to those in other reports [46]. On the other hand, our findings also seem to be at odds with some studies in which carotid artery calcifications were found to range from 1.5 to 5.7% [6, 8]. The relatively higher prevalence in our sample may be attributed to variations in the age groups and the FOVs used. Referral for further investigation and/or management was recommended.

**Figure 10:**
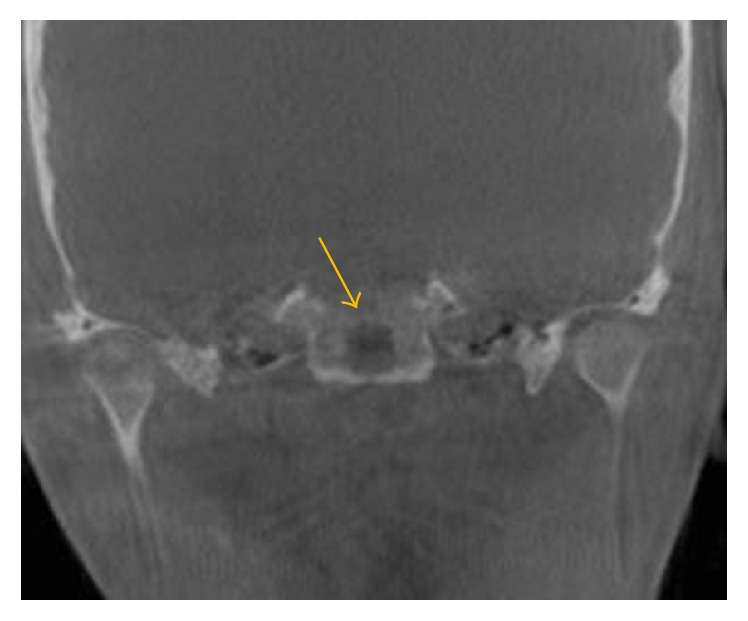
Coronal view demonstrates lytic lesion in the clivus.

We found one case of a lytic clivus lesion (0.15%) in a 13-year-old girl who underwent CBCT imaging for orthodontic treatment planning purposes (Figure 10). Based on incidental finding in the CBCT study and consensus arriving at by board-certified maxillofacial radiologists and neuroradiologists, MRI was recommended to further evaluate the lesion of interest. 3 T MRI of brain was performed. T1 and T2, pre- and postgadolinium images, were obtained. The examination demonstrated an enhancing lesion within the body of the clivus with homogenous signal intensity. Presence of cerebrospinal fluid was not observed within the lesion. Based on radiographic appearance, primary differential diagnosis of notochordal remnants was made. However, the possibility of chordoma cannot be ruled out. Further follow-up records are not available to obtain the definite diagnosis of the lesion.

We found 5 cases with lymph node calcifications with a mean age of 63 years. Cervical lymph node calcification is rare. It may suggest a limited differential diagnosis that includes chronic granulomatous conditions such as tuberculosis, sequela of radiation treatment, current or prior parasite infestation, prior treatment for lymphoma, and/or concurrent metastatic thyroid carcinoma, adenocarcinoma, or squamous cell carcinoma [12, 47].

MDCT or ultrasound is recommended if the presence of cervical lymph adenopathy is suspected [48]. One patient had longus colli muscle calcification. Patients with longus colli calcification may be asymptomatic or may present with either acute or chronic pain. Referral to the patient's physician for further investigation or treatment was recommended.

Radiology residents worked closely with the attending faculty who read the cases while tabulating the findings in this retrospective analysis. Any confounding signals in studies evaluated were immediately brought to the attention of board-certified oral and maxillofacial radiology and neuroradiology experts for further discussion and consensus decision. No additional findings were noted. It is thus recommended that all medium to large FoV CBCT and any limited area FoV studies be interpreted by board-certified oral and maxillofacial radiologists to adequately report findings and determine the best course of management for all categories of findings, incidental or otherwise. Collaborative efforts with neuroradiologists are central to patient management and referral. All studies must be carefully evaluated and reported. Prevalence of incidental findings requiring further investigations, imaging or otherwise, and appropriate intervention, as reported in our study and other studies is high enough to warrant reporting of CBCT studies by board-certified maxillofacial radiologists.

## 5. Conclusions

Our results show that large FoV CBCT studies may demonstrate a relatively high incidence of incidental findings in the head and neck region and outside of the maxillofacial skeleton, which may or may not require referral to medical practitioners, follow-up with additional complex imaging or other diagnostic tests, and/or surgical or nonsurgical intervention. The anatomic areas examined in this study are conventionally considered to be outside the regions of interest and expertise of dental clinicians. Presence of critical findings that need intervention prior to initiation of dental treatment demands careful and systematic evaluation of the entire volume by board-certified oral and maxillofacial radiologists. This study, unlike other previously reported CBCT studies, did not include incidental findings of the maxillofacial skeleton or the dentition. Often, detection of incidental findings may be challenging as the presentation could be subtle, requiring specific postprocessing of images to tease them out. This study highlights the importance of reviewing CBCT volumes in their entirety in order for clinically significant findings requiring referral and/or monitoring to be diagnosed and managed appropriately. Care must be coordinated with medical radiologists and physicians in a timely fashion.

## Figures and Tables

**Table 1:** Indications for CBCT examination.

Diagnostic tasks	Number of patients	Percentage
Implants	152	38
Pathology	121	30
Orthodontics	96	24
TMJ	31	8
Total	400	100

**Table 2:** Frequency of incidental findings from 400 CBCT scans.

General category of incidental findings	Number of incidental findings	Incidental findings (%)
*Soft tissue*	*194*	*29.71*
Thyroid/triticeous calcification	106	16.24
Tonsilloliths	55	8.42
Stylohyoid ligament/stylomandibular ligament	33	5.05
*Intracranial*	*176*	*26.96*
Pineal gland	73	11.18
Intracranial vascular calcification	69	10.57
Choroid plexus	17	2.6
Petroclinoid	17	2.61

*Cervical vertebrae*	*131*	*20.06*
Osteoarthritis	107	16.38
Lytic lesion	2	0.31
Vertebral misalignment (antero/posterolisthesis)	6	0.91
Fusion	5	0.77
Prior surgery	2	0.31
Suspected hemangioma/pneumatocysts	9	1.38

*Airway*	*75*	*11.49*
Narrowing of pharyngeal airway	45	6.90
Adenoidal hypertrophy	23	3.52
Asymmetry of pharyngeal airway	7	1.07

*Other*		
Carotid artery calcification	68	10.41
Lymph node	5	0.77
Calcification in subcutaneous tissue	1	0.15
Calcific tendonitis of the longus colli muscle	1	0.15

*Skull base *	*2*	*0.3*
Post. comm. artery aneurysm	1	0.15
Suspected clivus lesion	1	0.15

**Table 3:** Clinical significance of the incidental findings.

General category of incidental findings	Incidental findings	Number of incidental findings (%)
*(I) Referral *		*205 (31.39)*
Carotid atherosclerosis	Intracranial vascular calcification	69 (10.57)
	Extracranial vascular calcification	68 (10.41)
Airway issues	Narrowing of pharyngeal airway	45 (6.90)
	Asymmetry of pharyngeal airway	7 (1.07)
Cervical vertebral lesions	Vertebral malalignment (antero/posterolisthesis)	6 (0.91)
	Fusion	5 (0.77)
	Lytic lesion	2 (0.31)
Skull base	Suspected chordoma	1 (0.15)
	PCA^*∗*^ Aneurysm	1 (0.15)
Longus colli muscle	Calcific tendonitis of the longus colli muscle	1 (0.15)

*(II) Monitoring or follow-up*		*116 (17.76)*
Cervical vertebrae	Osteoarthritis	107 (16.38)
	Pneumatocyst/hemangiomas	9 (1.38)

*(III) No referral or monitoring*		*332 (50.85)*
Soft tissue calcifications	Thyroid/triticeous calcification	106 (16.24)
	Tonsillitis	55 (8.42)
	Stylohyoid/stylomandibular ligament calcification	33 (5.05)
Airway issues	Adenoidal hyperplasia	23 (3.52)
Intracranial findings	Pineal gland calcification	73 (11.18)
	Petroclinoid calcification	17 (2.61)
	Choroid plexus calcification	17 (2.6)
Cervical vertebrae	Prior surgery	2 (0.31)
Lymph node	Lymph node calcification	5 (0.77)
Subcutaneous tissue	Calcification in subcutaneous tissue	1 (0.15)

^*∗*^Posterior communicating artery.
